# Susquehanna Dental Association

**Published:** 1868-02

**Authors:** M. D. L. Dodson

**Affiliations:** Secretary.


### CORRESPONDENCE.

---

*Susquehanna Dental Association.*

WILLIAMSPORT PA., *Jan*, 17, 1868.

At a regular semi-annual meeting of the Susquehanna Dental Association, held at Milton, Pa., January 15, 1868, the following resolution with reference to the course pursued by the Dental Vulcanite Co., towards the Dental profession, was unanimously adopted, and the co-operation of the profession throughout the country solicited, to the end that no further annoyance may arise from that source:

RESOLVED, That the members of this Association consider the action of the so-called Goodyear Dental Vulcanite Company irregular, unjust and an absolute imposition; and that, as a body, we will discard and discourage the use of hard rubber for dental purposes, and adopt such substitutes as we may severally prefer.

By order of the Association.

M. D. L. DODSON,
*Secretary.*

